# Development of methods for the genetic manipulation of *Flavobacterium columnare*

**DOI:** 10.1186/1471-2180-8-115

**Published:** 2008-07-11

**Authors:** Andrew M Staroscik, David W Hunnicutt, Kate E Archibald, David R Nelson

**Affiliations:** 1 Department of Cell and Molecular Biology, University of Rhode Island, Kingston, RI 02881, USA; 2 Department of Biology, St. Norbert College, De Pere, WI 54115-2099, USA

## Abstract

**Background:**

*Flavobacterium columnare *is the causative agent of columnaris disease, a disease affecting many freshwater fish species. Methods for the genetic manipulation for some of the species within the *Bacteroidetes*, including members of the genus *Flavobacterium*, have been described, but these methods were not adapted to work with *F. columnare*.

**Results:**

As a first step toward developing a robust set of genetic tools for *F. columnare*, a protocol was developed to introduce the *E. coli *– *Flavobacterium *shuttle vector pCP29 into *F. columnare *strain C#2 by conjugal mating at an efficiency of 1.5 × 10^-3 ^antibiotic-resistant transconjugants per recipient cell. Eight of eleven *F. columnare *strains tested were able to receive pCP29 using the protocol. pCP29 contains the *cfxA *and *ermF *genes, conferring both cefoxitin and erythromycin resistance to recipient cells. Selection for pCP29 introduction into *F. columnare *was dependent on *cfxA*, as *ermF *was found not to provide strong resistance to erythromycin. This is in contrast to other *Flavobacterium *species where *ermF*-based erythromycin resistance is strong. The green fluorescent protein gene (*gfp*) was introduced into *F. columnare *strains under the control of two different native *Flavobacterium *promoters, demonstrating the potential of this reporter system for the study of gene expression. The transposon Tn*4351 *was successfully introduced into *F. columnare*, but the method was dependent on selecting for erythromycin resistance. To work, low concentrations of antibiotic (1 μg ml^-1^) were used, and high levels of background growth occurred. These results demonstrate that Tn*4351 *functions in *F. columnare *but that it is not an effective mutagenesis tool due to its dependence on erythromycin selection. Attempts to generate mutants via homologous recombination met with limited success, suggesting that RecA dependent homologous recombination is rare in *F. columnare*.

**Conclusion:**

The conjugation protocol developed as part of this study represents a significant first step towards the development of a robust set of genetic tools for the manipulation of *F. columnare*. The availability of this protocol will facilitate studies aimed at developing a deeper understanding of the virulence mechanisms of this important pathogen.

## Background

The causative agent of columnaris disease is the bacterium, *Flavobacterium columnare *[1]. This fish disease is common in freshwater environments, affects numerous fish species [2], and is responsible for significant economic losses in the US channel catfish (*Ictalurus punctatus*) industry [3]. Virulence is known to vary between strains of *F. columnare *[4,5] and there is some evidence that strains vary in host preference [6]. Infected fish often exhibit external lesions on the body surface, gills and fins [2], but during some outbreaks bacteria can be isolated from moribund fish that exhibit no external signs of infection. *Flavobacterium columnare *is an opportunistic pathogen and is particularly problematic in commercial aquaculture facilities where high fish densities are required for profitability.

A substantial amount of work has been done to develop methods for the rapid identification of *F. columnare *during outbreaks [7,8] and in distinguishing between more and less virulent strains of the bacterium [6,9-13]. Efforts have also been made to understand the mechanisms of virulence employed by the organism. Several factors have been proposed, including the ability to adhere to surfaces [14-16], extracellular protease activity [17], and chondroitin AC lyase activity [12,18,19]. The bulk of the evidence for these factors playing a role in virulence is suggestive, based primarily on observed symptoms of the disease. Little work has been done to characterize the genetic basis of virulence due, in part, to the lack of a robust genetic system for the manipulation of this important pathogen. The ability to introduce foreign DNA into strains of *F. columnare *would greatly increase our ability to study mechanisms of virulence in this pathogen.

While no reports of the successful introduction of plasmids or transposons into *F. columnare *exist in the peer-reviewed literature, other members of the genus *Flavobacterium *have proven amenable to genetic manipulation. Expression of genes and replication of plasmids in members of the genus *Flavobacterium *required modifications of existing expression and mutagenesis vectors because systems optimized for the better-studied groups such as Proteobacteria do not function in Bacteroidetes [20,21]. The first successful mutagenesis of a member of this genus was reported by McBride and Kempf [21] for *Flavobacterium johnsoniae *with the introduction of the *Bacteroides *transposon Tn*4351 *[22] carrying the erythromycin resistance gene *erm*F. They also constructed an *E. coli*-*F. johnsoniae *shuttle vector by combining the pCU19-based suicide vector pLYL03 [23] with a cryptic plasmid (pCP1) isolated from *Flavobacterium psychrophilum *strain D12 [21]. The transposon has subsequently been shown to work in one *F. psychrophilum *strain [24] and the shuttle vector has been introduced into both *F. psychrophilum *[24] and *Flavobacterium hibernum *[25].

The successful introduction of these vectors into other *Flavobacterium *species led us to hypothesize that, under the proper conditions, *F. columnare *would be susceptible to genetic manipulation using the vectors and markers described above. The objective of this study was to determine the conditions required for *F. columnare *to accept DNA by conjugal mating and to begin exploring the potential of a green fluorescence protein (Gfp) based reporter system for the study of native *F. columnare *promoters.

## Results

### Introduction of pCP29 into *F. columnare*

The *E. coli *– *Flavobacterium *shuttle vector pCP29 was introduced into *F. columnare *strain C#2 by conjugation with *E. coli *S17-1 at a frequency of 1.5 × 10^-3 ^cefoxitin-resistant transconjugants per recipient cell. Attempts to extract plasmids from *F. columnare *cultures with commercial kits resulted in low yields. As a result, the presence of the plasmids in *F. columnare *strains was confirmed two ways. First, the cefoxitin gene was amplified by PCR with primers pr32 and pr33 using both the low yield plasmid extractions and genomic DNA extracted from cefoxitin resistant *F. columnare *strains as the template. Genomic DNA from the cefoxitin sensitive *F. columnare *parental strain was used as the negative control. In the second approach, the plasmid was reintroduced back into *E. coli *cells by electroporation using the low yield plasmid extractions as the source of the DNA in the transformation protocol. The recovery of the plasmid from these *E. coli *cells, demonstrated its presence in the cefoxitin resistant *F. columnare *strains.

Ten μg ml^-1 ^of cefoxitin was sufficient to prevent background growth as all cefoxitin resistant colonies tested were found to harbor the plasmid. In total, eight of eleven *F. columnare *strains screened took up pCP29 by conjugal transfer. The efficiency of the transfer was not estimated for any strains other than C#2, but based on the number colonies seen on the selection plates, two of the strains (1191-B and 94-078) appeared to take up the plasmid at an efficiency lower than that achieved with C#2. The other 6 produced transconjugants at rates similar to C#2 (Table 1). The virulence to channel catfish of 10 of the 11 strains used has been previously reported [13,26]. All 6 of the more virulent strains were capable of taking up pCP29. Of the 4 less virulent strains, 2 took up the plasmid and 2 did not (Table 1). The virulence of Fc14-56 to channel catfish is not known, but it is capable of causing disease in zebra fish (*Danio rerio*) [27].

**Table 1:** Ability of *F. columnare *strain to receive pCP29 by conjugation with *E. coli *S17-1.

**Strain**	**Results of mating attempts**^a^	**Virulence in channel catfish**
C#2	++	High
AL-203-94	++	High
Fc14-56	-	Unknown
94-060	++	High
1191-B	+	High
94-078	+	High
94-081	++	High
90-059	-	Low
L90-659	++	Low
92-002	-	Low
C91-20	++	Low

^a^Transconjugates isolated at an efficiency equivalent to that achieved with C#2: ++; Transconjugants isolated but at low efficiency: +; no transconjugants isolated: -.

pCP29 containing transconjugants were also obtained using erythromycin selection, but for growth to occur, the erythromycin concentration had to be lowered to 1 μg ml^-1^. This resulted in high background growth, indicating that the erythromycin resistance gene *ermF *does not impart strong resistance to *F. columnare*. Also, the *E. coli *donor strain was not inhibited by 1 μg ml^-1 ^of erythromycin, necessitating the use of 1 μg ml^-1 ^tobramycin for counter selection against the *E. coli*. Filters for conjugation were incubated on *Flavobacterium columnare *Growth Medium (FCGM), Ordal's, and Modified Ordal's (MO) plates, and transconjugants were only isolated when FCGM plates were used for this step.

### Expression of *gfp *in *F. columnare*

**Figure 1:**
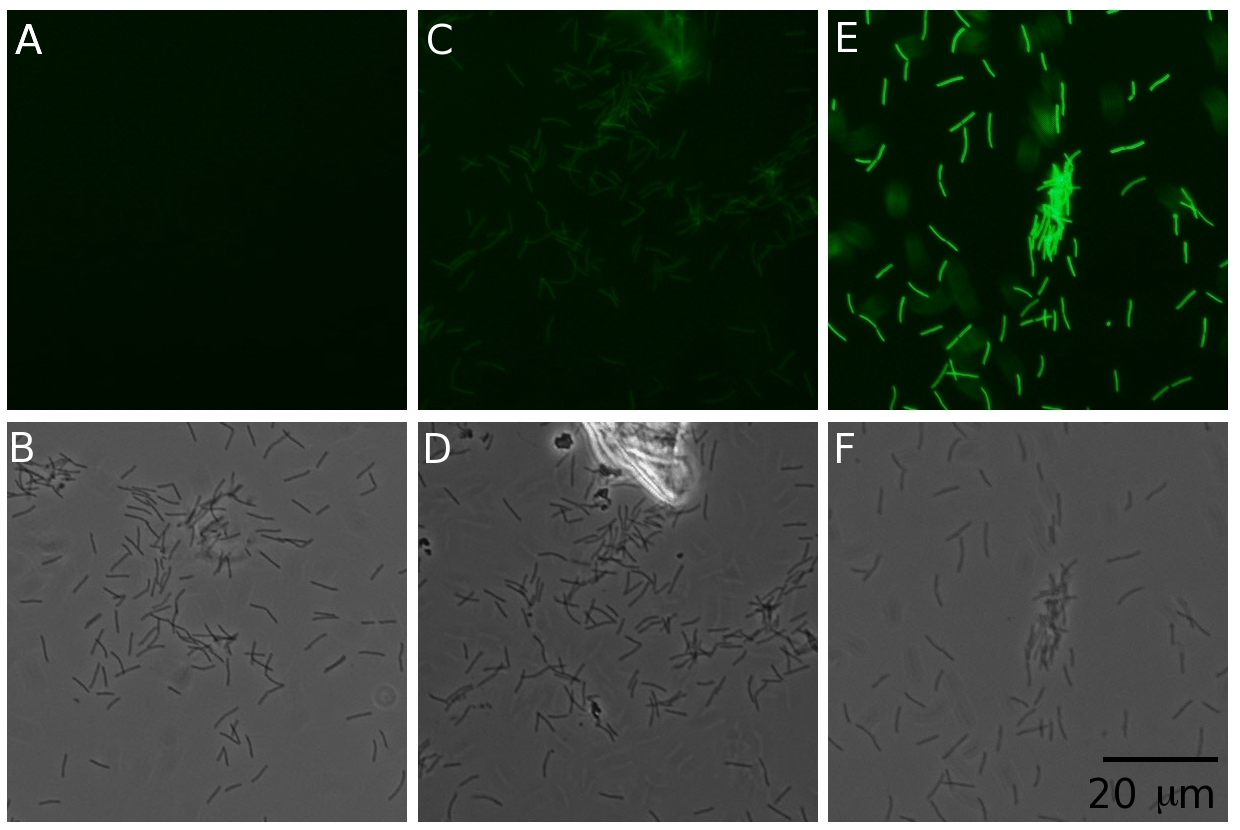
**Demonstration of Gfp expression levels in *F. columnare *strain C#2 containing plasmids pAS29 (A and B) pAS36 (C and D) and pAS43 (E and F) using epifluorescence (A, C and E) and transillumination/phase contrast (B, D and F) microscopy**. The same field is shown for epifluorescence and phase contrast micrographs for each strain. Exposure was varied in the pictures using transillumination to optimize each image, but for comparative purposes the excitation energy and image exposure times were held constant in the three epifluorescence images. All six panels are drawn to the same scale.

Introduction of the Gfp gene into *F. columnare *strain C#2 under control of the *map *promoter on plasmid pAS36 resulted in expression of the gene at levels that could be detected by both a fluorescence plate reader and by epifluorescence microscopy (Figure 1c). This result demonstrates that *gfp *expression can be used to detect and quantify expression of native *F. columnare *genes.

To increase the level of expression, the recently described strong promoter from the *F. johnsoniae ompA *gene [28] was also placed in front of *gfp *in pAS29 creating pAS43. pAS43 was introduced into *F. columnare *strain C#2. The resulting fluorescence was greater in cells containing *gfp *driven by the *ompA *promoter than in cells containing *gfp *driven by the *map *promoter (Figures 1c and 1e). The difference in Gfp fluorescence was quantified using the fluorescence plate reader. Gfp fluorescence values and standard errors of the mean were 41 ± 0.64, 211 ± 26 and 3,085 ± 22 for strain C#2 containing plasmids pAS29 (no promoter), pAS36 (*map *promoter) and pAS43 (*ompA *promoter) respectively. The significance of the differences in fluorescence levels detected between strains was assessed using paired t-tests on log-transformed data. After adjusting for multiple tests, all differences were found to be significant with p-values less than 0.0001.

### Mutagenesis attempts using Tn*4351*

**Figure 2:**
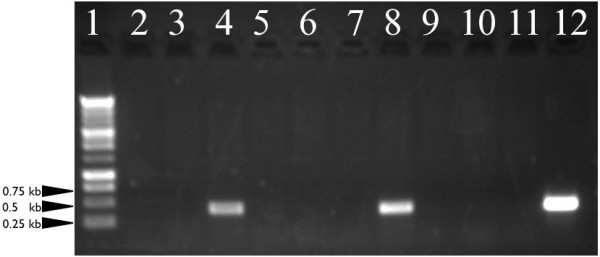
**Demonstration of *Tn*4351 transposable element integration into the genome of *F. columnare *strain AL-203-94 following conjugative mating**. Primers pr54 and pr56 targeting a 435 bp fragment of the *tetX *gene contained within the transposon were used to screen for the presence of the transposon in *F. columnare *genomic DNA. PCR products were run on a 1% agarose gel at 80 V for 45 min and visualized after staining with ethidium bromide. Lane 1: 1 kb ladder, markers range from 250 to 10,000 bp; Lanes 2–11: PCR product from genomic DNA extracted from colonies that grew on an Ordals agar plate augmented with 1 μg ml^-1 ^of erythromycin and Lane 12: Tn*4351 *containing plasmid pEP4351 (positive control).

Transposon mediated random mutagenesis was performed using the *Bacteroides *transposon Tn*4351 *[22]. Tn*4351 *contains the erythromycin resistance gene *ermF*, necessitating the use of erythromycin as the selective marker. As with efforts to use erythromycin to introduce pCP29 into *F. columnare*, antibiotic concentrations of 1 μg ml^-1 ^or lower were required for any growth to occur. At these low concentrations, a significant amount of background growth was observed. Transposon mutagenesis was attempted in three strains (C#2, AL-203-94 and Fc14-56) and Tn*4351 *was successfully introduced into *F. columnare *strain AL-203-94. Only two of ten colonies isolated from plates containing 1 μg ml^-1 ^erythromycin contained the transposon (Figure 2). While the two identified insertions demonstrate that the transposon is capable of integrating into the *F. columnare *genome, the high number of false positives suggests that this *ermF *based transposon is not a useful tool for the generation of mutants in this organism.

### Insertion mutagenesis by homologous recombination

Several attempts to make mutants by homologous recombination with the *ermF *containing suicide plasmid pLYL03 [23] were unsuccessful. No colonies appeared at erythromycin concentration greater than 1 μg ml^-1^, and significant background growth occurred below this concentration (data not shown).

**Figure 3:**
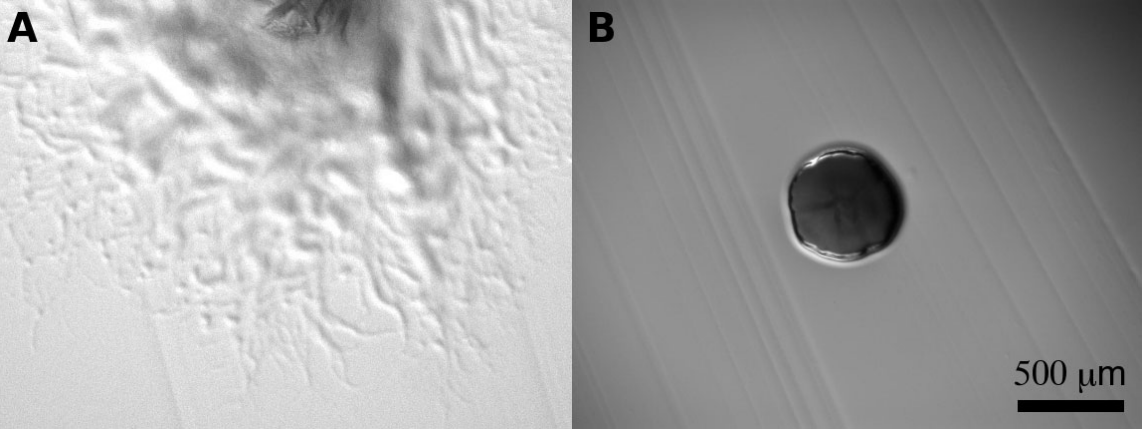
**Photomicrograph of *F. columnare *colonies**. Colonies were grown for 2 days at 27°C on Ordal's agar medium. (A) Wild-type *F. columnare *C#2. (B) *gldJ *knockout mutant FcAS44. Both panels are drawn to the same scale.

A cefoxitin based *F. columnare *suicide vector, pAS42, was created by replacing *Flavobacterium *replicative functions of pCP29 with a truncated *gldJ *sequence as described in Methods. Using the mating protocol described below, pAS42 was introduced into C#2 resulting in the successful isolation of non-motile, cefoxitin resistant colonies (Figure 3). Mutants were isolated at an efficiency of roughly 1 × 10^-6 ^cefoxitin-resistant mutants per recipient cell. This is 1,000-fold lower than the rate at which the pCP1 based shuttle vector, pCP29, can be introduced to strain C#2. Disruption of *gldJ *was confirmed by PCR amplification and sequencing of the novel junction formed by the insertion of the mutagenesis vector in to the *gldJ *gene. PCR was done using primers pr88 and pr93. Sequencing across the novel junction was accomplished from both directions using primers pr88 and pr104 (data not shown).

## Discussion

### Conditions for conjugal plasmid transfer from *E. coli *to *F. columnare*

While some members of the genus *Flavobacterium *have proven amenable to receiving plasmids via conjugal mating [21,24,25,29], no reports exist of the introduction of plasmids into *F. columnare*. Here we report the first successful introduction of plasmids into *F. columnare *using vectors developed from the *F. psychrophilum *cryptic plasmid pCP1 [21]. These results extend the host range of pCP1-based shuttle vectors to *F. columnare*.

Several factors appear to contribute to the successful transfer of plasmids from *E. coli *to *F. columnare*. One is the use of culture conditions for the initial growth of *F. columnare *that allow the cells to grow to relatively high cell density with minimal clumping or biofilm formation. Numerous media have been described that support the growth of *F. columnare *[30,31], but MO was chosen for the initial growth step due to the rapid growth and minimal biofilm formation observed with the use of this medium. While transconjugants were obtained from cultures grown in both Ordal's medium and FCGM, MO was deemed superior because of problems with low cell density, cell clumping, and biofilm formation with Ordal's medium. Cell clumping was not a problem with FCGM, but not all strains grew to a high cell density in this medium.

A more important part of the mating protocol was the medium used for the conjugal mating step itself. Ordal's, MO and FCGM plates were all tested for the incubation of the mating filters, but transconjugants were isolated only when FCGM plates were used. In conjugal mating protocols developed for other *Flavobacterium *species, the concentrated mixtures of donor and recipient cells are spotted onto the mating plates directly [21,24,25]. With *F. columnare*, the use of 47 mm diameter 0.45 μm pore size nitrocellulose filters was necessary because the tightly adhering mass of cells was difficult to remove from the agar surface, but could easily be scraped from the surface of the filter.

The conjugation efficiency of 1.5 × 10^-3 ^cefoxitin-resistant transconjugants per recipient cell using pCP29 is greater than what has been reported for *F. psychrophilum *[24] and roughly equivalent to the highest rates reported for *F. johnsoniae *[21]. The fact that eight of eleven *F. columnare *strains screened took up pCP29 suggests that this protocol can be used with many of the virulent strains of *F. columnare *available for study, although rates of uptake varied between strains and two strains did not take up the plasmid under the conditions tested (Table 1). This is in contrast to the method developed for *F. psychrophilum *where only one strain has been shown to be capable of accepting the plasmids, [24] possibly owing to differential DNA methylation mechanisms or plasmid incompatibility.

### Expression of *gfp *in *F. columnare*

*Flavobacterium columnare *cells must respond to varying environments over the course of the infection process. These include areas on the external and internal surfaces of the fish as well as the surrounding environment. For example, studies using mucus scraped from the surface of Atlantic salmon (*Salmo salar L.*) [32] suggest that *F. columnare *regulate both biofilm production and extracellular protease activity in response to exposure to fish mucus. The mechanism of dispersal of *F. columnare *through the host from initial, local sites of infection is also unclear. Studies of the response of *F. columnare *to changing environmental conditions would be aided by Gfp-expressing strain, which would allow the direct visualization of either biofilm formation or the infectious process by *F. columnare*.

For such a strain to be useful, Gfp-expression levels must be high enough for easy visualization. Promoters that drive gene expression in other gram-negative bacteria generally do not function well in the Bacteriodetes [33], including *Flavobacterium *species [21,25]. In *Bacteroides fragilis*, analysis of housekeeping genes led to the description of two consensus regions -7/-33 with the following motifs: TAnnTTTG/TTTG [20]. Recently, Chen et al. [28] described a strong promoter from the *ompA *gene of *F. johnsoniae *that contained these two consensus motifs and led to high levels of fluorescence when used to drive *gfp *expression. Mutation analysis was also used to describe a putative ribosomal binding site (RBS) consensus sequence: TAAAA found 2 to 12 bases from the gene start codon [28].

The successful introduction of pCP29 into *F. columnare *led to an evaluation of the shuttle vector as a tool for the study of gene expression. To explore this potential, a promoterless copy of the GFPmut1 gene [34] was cloned into the *Kpn*I-*Pst*I sites of pCP29 creating pAS29. The *Kpn*I restriction site was positioned just upstream of the beginning of the *gfp *gene. This arrangement allowed for the placement of different promoters upstream of *gfp*.

In this study, two promoters were assessed. The first was the recently described *F. johnsoniae *strong promoter P_ompA _[28]. The second promoter evaluated was the region upstream of *map*, a gene which codes for a membrane associated metalloprotease in *F. columnare *[35]. The promoter region of this gene was chosen because protease activity is a proposed virulence factor [17] and real-time RT-PCR analysis suggests that the gene is constitutively expressed in *F. columnare *(Staroscik and Nelson unpublished data).

The P_ompA _region contains all three of the consensus motifs (-33, -7, RBS) described above, while the native *F. columnare *promoter *map *contains the RBS and -7 motifs but not the -33 TTTG motif. The substantial increase in Gfp fluorescence driven by the *ompA *promoter (P_ompA_) relative to the *map *promoter (P_map_) is consistent with the findings of others that while the -33, TTTG motif is not essential for gene expression, it is necessary for full activity [20]. The presence of a native promoter in *F. columnare *lacking the -33 consensus sequence suggests that the absence of this motif is a strategy used by the organism to drive low level constitutive expression of some genes. Gene expression studies using constructs such as pAS36 and pAS43 should facilitate the study of gene expression under environmentally relevant conditions and the results with the *map *promoter suggest that *gfp *expression can be used in the study of moderately expressed *F*.*columnare *promoters. The availability of a plasmid containing the *gfp *gene linked to a strong promoter should also open the door to studies involving the direct observation of live cells under a variety of conditions such as on the surface of fish or *in vivo *during the infection process.

### Transposon and site-specific homologous recombination mutagenesis in *F. columnare*

Three resistance markers have been used for the genetic manipulation of *Flavobacterium *species: The erythromycin resistance gene *ermF*, the tetracycline resistance gene *tetQ*, and the cefoxitin resistance gene *cfxA*. The cloning vectors pCP11, pCP23, pCP29, pEP4351 and pLYL03 all contain *ermF *[21,23,36,37]. In addition to *ermF*, pCP23 and pCP29 contain *tetQ *[36] and *cfxA *[37] respectively. While *ermF *has been found to impart strong resistance to other *Flavobacterium *species [21,24,25], the *F. columnare *strains tested in this study remained sensitive to erythromycin after introduction of *ermF *containing plasmids. The reason(s) for the poor performance of *ermF *in *F. columnare *is not known. It seems unlikely that promoter strength is the issue since the region upstream of the *ermF *gene contains the strong promoter -7/-33 consensus sequence [21,24,25]. The poor performance of *ermF *suggests that existing *Flavobacterium *vectors will need to be modified for use in *F. columnare*.

The successful introduction of Tn*4351 *into *F. columnare *strain AL-203-94 demonstrates that existing transposon-based mutagenesis systems function in *F. columnare*. Nevertheless, the high level of background growth due to the low erythromycin levels required for growth suggests that the existing transposon will need to be modified by the addition of another resistance marker before it is an effective tool for the study of this organism. The modification of the transposon and the identification of additional antibiotic resistance genes functional in *F. columnare *should be a high priority for future work.

Difficulty associated with high background growth was also experienced with attempts to use the *ermF *based site directed mutagenesis vector pLYL03 to knock out specific genes by homologous recombination. This led us to construct a new *cfxA *based vector by removing the *Flavobacterium *origin of replication from pCP29. This construct was used to isolate *gldJ*^- ^motility mutants. While this effort was successful, multiple mating attempts were required before cefoxitin resistant, non-motile mutants were identified. Subsequent efforts to disrupt other genes by this approach have been successful, but the process was inefficient, requiring multiple attempts before mutants were isolated (Staroscik and Nelson unpublished data). Given the efficiency with which pCP29 can be introduced into *F. columnare*, these results suggest that homologous recombination events are rare. This is consistent with work in *F. johnsoniae *where insertion mutants of some genes have been made by homologous recombination [36], but the efficiencies have been quite low (Hunnicutt and McBride personal communication) and attempts with some genes have not succeeded [38].

In *E. coli*, the major homologous recombination pathway is dependent on the activity of the genes *recA*, *recB*, and *recC *[39-41]. The recently sequenced genomes of *F. psychrophilum *[42] and *F. johnsoniae *(accession number CP000685; unpublished data) reveal that while both contain *recA*, neither contain *recB *or *recC*. The absence of these genes is not unique to *Flavobacterium *[43], but their absence may be part of the reason homologous recombination events are rare in members of this genus. Complementation of the motility mutant has yet to be accomplished, demonstrating further the need to develop additional selectable markers and cloning vectors for members of the genus *Flavobacterium*.

## Conclusion

The lack of robust methods for the genetic manipulation of *F. columnare *represents a substantial barrier to understanding virulence mechanisms in this important fish pathogen. The availability of the conjugation protocol described in this study will facilitate work aimed at deepening of our understanding of the virulence mechanisms of *F. columnare*. While conditions for efficient random mutagenesis still need to be resolved, the methods described in this report represent a significant first step towards the development of a robust set of genetic tools for *F. columnare*. In addition to the method for introduction of foreign DNA into *F. columnare*, the new Gfp-based reporter constructs should facilitate studies of gene expression and *in vivo *cell localization.

## Methods

### Bacterial strains and plasmids

The bacterial strains and plasmids used in this study are listed in Table 2. *Escherichia coli *were routinely grown in LB broth or plates made without the glucose [44] at 37°C. To optimize mating conditions, *F. columnare *strains were grown at 27°C on a variety of media (Table 3). Liquid cultures were shaken at 220 rpm. For *E. coli*, ampicillin was used at a concentration of 200 μg ml^-1 ^and chloramphenicol was used at 10 μg ml^-1^. For *F. columnare*, cefoxitin was used at 10 μg ml^-1^, erythromycin at 1 μg ml^-1^, and tobramycin at 1 μg ml^-1 ^(for counter selection against *E. coli*, when needed).

**Table 2:** Strains and plasmids used in this study

**Strain or plasmid**	**Genotype or description**	**Source or reference**
Bacterial Strains		

*E. coli*		

S17-1	*hsd*R17 (r_k_^- ^m_k_^-^)*rec*A RP4-2(Tc^r^::Mu-Km^r^::Tn7 Str^r^)	
TOP10	F^- ^*mcr*A Δ(*mrr-hsd*RMA-*mcr*BC) φ80*lac*ΔM15 Δ*lac*X74 *rec*A1 *ara*D139 Δ(*ara-leu*)7697 *gal*U *gal*K *rps*L (Str^r^) *end*A1 *nup*G	Invitrogen

*F. columnare*		

C#2	Wild Type	[13]
AL-203-94	Wild Type	[13]
Fc14-56	Wild Type	[26]
94-060	Wild Type	[26]
1191-B	Wild Type	[26]
94-078	Wild Type	[26]
94-081	Wild Type	[26]
90-059	Wild Type	[26]
L90-659	Wild Type	[26]
92-002	Wild Type	[26]
C91-20	Wild Type	[26]
FcAS44	*gldJ *knockout mutant of C#2	This Study

Plasmids		

pAMSTA39	PCR cloning vector with promoter-less *gfp*; Ap^r ^Km^r^	This Study
pAS29	Promoter-less *gfp* containing *E. coli-Flavobacterium *shuttle vector; Ap^r ^(Em^r^, Cf^r^)	This Study
pAS36	P_map_-*gfp* containing *E. coli-Flavobacterium *shuttle vector; Ap^r ^(Em^r^, Cf^r^)	This Study
pAS42	1400-bp fragment of *gldJ *in pCP29; Ap^r ^(Em^r^, Cf^r^)	This Study
pAS43	P_ompA_-*gfp* containing *E. coli-Flavobacterium *shuttle vector; Ap^r ^(Em^r^, Cf^r^)	This Study
pCE320	*gfp*-containing *E. coli-Borrelia burgdorferi *shuttle vector; Ap^r^	[46]
pCR4-TOPO	PCR cloning vector; Ap^r ^Km^r^	Invitrogen
pCP11	*E. coli – Flavobacterium *shuttle plasmid; Ap^r ^(Em^r^)	[21]
pCP29	*E. coli – Flavobacterium *shuttle plasmid; Ap^r ^(Cf^r ^Em^r^)	[37]
pEP4351	λpir dependent R6K oriV; RP4 oriT; Cm^r ^Tc^r ^(Em^r^); Tn4351 mutagenesis vector	[53]
pCR4-TOPO	PCR cloning vector; Ap^r ^Km^r^	Invitrogen

**Table 3:** Media used in this study

**Ingredients (g L^-1^)**	**Ordals/Cytophaga (Ord)**^a^	**Modifled Ordals (MO)**^b^	**FCGM**^c^
Tryptone	0.5	0.5	8.0
Beef extract	0.2	0.2	
Yeast extract	0.5	0.5	0.8
NaCl		1.76^d^	5.0
Na_2_SO_4_		0.147^d^	
NaHCO_3_		0.008^d^	
KCl		0.025^d^	
KBr		0.004^d^	
MgCl_2 _× 6 H_2_O		0.187^d^	
MgSO_4 _× 7 H_2_O			1.0
CaCl_2 _× 2 H_2_O		0.041^d^	0.74
SrCl_2 _× 6 H_2_O		0.0008^d^	
H_3_BO_3_		0.0008^d^	
Sodium acetate	0.2	0.2	
Sodium citrate			1.5
Agar (for plates)	10	10	10

^a^Ordal and Rucker [54]

^b^This study. ^c ^Farmer [30]

^d^Salts were mixed in a 10× stock as NSS after Marden et al. [51]

### Bacterial mating

The *E. coli *donor strain used for conjugal transfer was S17-1. For bacterial mating, both donor and recipient cells were grown to mid-log phase, concentrated by centrifugation (5,500 × *g*, 10 min), washed once with modified Ordal's (MO) and resuspended in either MO (recipient cells) or a 1:1 mixture of MO and 10 mM MgSO_4 _(donor cells). Concentrated donor and recipient cells were mixed at a ratio of 1:1 based on OD_600 _readings obtained prior to concentrating. The mixture was vacuum filtered onto a 0.45 μm pore-sized nitrocellulose membrane filter (Fisher Scientific, Suwanee, GA). The filter was then placed face up on an FCGM agar plate and incubated over night (18–20 h) at 27°C. Following incubation, the cells were scraped off the filter, resuspended in MO broth, and the suspension homogenized with a 1 ml syringe and a 27 gauge needle. The homogenized suspension was spread on Ordal's plates containing 10 μg ml^-1 ^of cefoxitin to select for transconjugants. Plasmid-containing *F. columnare *colonies became visible after 48 h of incubation at 27°C.

### DNA isolation, amplification, and electrophoresis

Kits and enzymes were used following the manufacturer's instructions. Genomic DNA was extracted from 10 ml of *F. columnare *cultures grown for 16 h in MO using the Qiagen DNeasy tissue kit (Qiagen, Valencia CA). Plasmids were isolated from the relevant *E. coli *strains with QIAprep Spin Miniprep kit. PCR was performed with the Qiagen Taq PCR Master Mix Kit. A typical PCR reaction contained the Qiagen kit components plus 50 to 100 ng of template DNA and 100 nM of each primer. PCRs were run for 25 cycles. Elongation time was calculated as 1 min per kilobase of amplification product length. Annealing temperatures were varied according to the primer melting temperatures. Primers used in this study are listed in Table 4. Agarose gel electrophoresis was performed using standard techniques [45]. DNA sequencing was performed at the University of Rhode Island Genomics and Sequencing Center.

**Table 4:** Primers used in this study

**Primer**	**Sequence**^a^
pr26	5'-GCTAGGTACCATTTTTACTTTTTAGTGTTTCTATAAAAG-3'
pr32	5'-CCCGAAGCAGGGTTATGCAGCGGAAAAATT-3'
pr33	5'-GCCGATTGCCGACTGGTTCAGGGAGCAAT-3'
pr35	5'-GCTAGGTACCTCGAGCCTGTACCCATAAGATTAATACTAAATAA-3'
pr37	5'-GCTAGGTACCATGAGTAAAGGAGAAGAACTTTTCAC-3'
pr38	5'-GCTAGCTGCAGCAGATCTATTTGTATAGTTCATCCA-3'
pr44	5'-GGTACCGGCAGCGCATACCAAAGAACACTTAGACAAGGCA-3'
pr45	5'-GCTAGGTACCTTTTTAATTACAATTTAGTTAATTACAAGCAAAA-3'
pr46	5'-GCTAGCCCGGGCACGATTGGAATAACACTCCATCTCAGC-3'
pr47	5'-GCTAGCATGCACCTACGCGAGACATAGCACATCT-3'
pr54	5'-TTGGTGGTGGACCCGTTG-3'
pr55	5'-GCTGTTTCACTCGGTTTATTCTCA-3'
pr56	5'-ATCACCTTCACCCTCTCCACTGAC-3'
pr88	5'-TTAATGCAGCTGGCACGACAGGTT-3'
pr93	5'-AAACATTTCCCTCCTTAT-3'
pr104	5'-ACCTACTGAAAGTATGAAAGTAAAC-3'

^a^restriction sites on primers are underlined

### Construction of the pCP29 gfp expression vector

A promoterless copy of the green fluorescent protein gene (*gfp*) was amplified from the plasmid pCE320 [46] with the forward primer pr37 containing a *Kpn*I site and the reverse primer pr38 containing a *Pst*I site. The PCR fragment was cloned into pCR4-TOPO vector (Invitrogen, Carlsbad, CA) using electrocompetent TOP10 cells, creating plasmid pAMSTA39. pAMSTA39 was cut with *Kpn*I and *Pst*I and the *gfp *fragment gel purified using the Qiagen QIAEX II Gel Extraction Kit. The *Kpn*I/*Pst*I fragment was ligated into pCP29 which had been cut with the same enzymes creating plasmid pAS29 (Table 2). All ligations were performed using T4 DNA ligase (Promega, Madison, WI) according to the instructions of the manufacturer.

The promoter region of the membrane associated protease gene *map *[35] was PCR amplified from genomic DNA isolated from *F. columnare *strain C#2 using primers pr26 and pr35 both containing *Kpn*I sites. Primer pr35 also contained an *Xho*I site to allow restriction analysis of the promoter orientation in the final construct. The PCR fragment was cleaned using the Qiagen QIAquick PCR Purification Kit and ligated into plasmid pAS29 that had also been cut with *Kpn*I and treated with calf intestinal alkaline phosphatase (CIAP; Promega), according to the instructions of the manufacturer, creating plasmid pAS36. This construct contains *gfp *driven by the *map *promoter.

A second pCP29 based *gfp *construct was created by placing the *ompA *promoter from *F. johnsoniae *[28] in front of the *gfp *gene in pAS29. This was done using the primers pr44, pr45, genomic DNA from *F. johnsoniae *strain UW101 (NCBI Taxonomy ID 376686) and the procedure described above. This construct, pAS43, contains *gfp *driven by the *ompA *promoter. The nucleotide sequence of the promoter regions of pAS36 and pAS43 was confirmed by sequencing with primer pr56

### Construction of a pCP29 based suicide vector

The *E. coli*-*Flavobacterium *shuttle vector containing the cefoxitin resistance gene *cfxA *was converted into a homologous recombination-insertional mutagenesis vector by the removal of the pCP1 fragment containing the origin that allows the plasmid to replicate in *Flavobacterium *species. This was accomplished by cutting pCP29 with the restriction enzymes *Sma*I and *Sph*I and isolating the 8,100 bp fragment by gel purification. The gene chosen for insertion mutagenesis by homologous recombination was the motility gene *gldJ *[47]. Primers were designed using Genbank sequences with accession number AAV52895. A 1,400 bp fragment of the *gldJ *gene was amplified by PCR from *F. columnare *strain C#2 genomic DNA using primers pr46 and pr47 containing *Sma*I and *Sph*I sites respectively (Table 4). The PCR fragment was cleaned and cut with *Sma*I and *Sph*I sites and ligated into the 8,100 bp fragment isolated from pCP29. This resulted in the plasmid pAS42 (Table 4).

### Microscopy

For phase contrast microscopy, wet mounts using 5 to 10 μl of cultures were photographed using the ZEISS Axioplan 2 Imaging System at the University of Rhode Island Genomics and Sequencing Center [48]. Epifluorescence microscopy was performed using the same system with the FITC filter set. Micrograph images were processed using the open source programs ImagJ [49] and The GIMP [50].

### Quantitative analysis of Gfp production

Gfp expression was measured in 50 ml cultures of *F. columnare *grown at 27°C shaking for 20 hr in MO. Culture were concentrated 20-fold by centrifugation (5,500 × *g*, 10 min) and resuspended in a 10% concentration of nine-salt solution, (NSS; a carbon-, nitrogen-, and phosphorus-free salt solution) [51]. Fluorescence was measured in 200 μl aliquots in a Spectra Max M2 plate reader (Molecular Devices, Sunnyvale CA) with an excitation wavelength of 485 nm and an emission wavelength of 538 nm. All experiments were performed with four replicates. The significance of differences in expression levels between strains were assessed with paired t-tests on log transformed data. Significance levels were adjusted for multiple tests using the Bonferroni method [52].

## Authors' contributions

AMS, DWH and DRN conceived of the study. AMS developed the mating protocol; designed and constructed the plasmids generated for the study; performed the microscopic analysis; and drafted the manuscript. DWH participated in the development of the mating protocol and edited the manuscript. KEA participated in the design and construction of the plasmids generated for the study and screened multiple *F. columnare *strains for the ability to accept pCP29 by conjugal mating. DRN supervised the work and edited the manuscript. All authors read and approved the final manuscript.

## References

[B1] Bernardet J-F, Segers P, Vancanneyt M, Berthe F, Kersters K, Vandamme P (1996). Cutting a Gordian knot: emended classification and description of the genus *Flavobacterium*, emended description of the family *Flavobacteriaceae*, and proposal of *Flavobacterium hydatis *nom. nov. (basonym, *Cytophaga aquatilis *Strohl and Tait 1978). Int J Syst Bacteriol.

[B2] Austin B, Austin DA (1999). Bacterial fish pathogens: diseases in farmed and wild fish.

[B3] USDA (2003). Catfish 2003 Part II: Reference of Foodsize Catfish Health and Production Practices in the United States, 2003.

[B4] Decostere A, Haesebrouck F, Devriese LA (1998). Characterization of four *Flavobacterium columnare *(*Flexibacter columnaris*) strains isolated from tropical fish. Vet Microbiol.

[B5] Shoemaker CA, Olivares-Fuster O, Arias CR, Klesius PH (2008). *Flavobacterium columnare *genomovar influences mortality in channel catfish (*Ictalurus punctatus*). Vet Microbiol.

[B6] Olivares-Fuster O, Baker J, Terhune J, Shoemaker C, Klesius P, Arias C (2007). Host-specific association between *Flavobacterium columnare *genomovars and fish species. Syst Appl Microbiol.

[B7] Bader JA, Shoemaker CA, Klesius PH (2003). Rapid detection of columnaris disease in channel catfish (*Ictalurus punctatus*) with a new species-specific 16-S rRNA gene-based PCR primer for *Flavobacterium columnare*. J Microbiol Meth.

[B8] Welker TL, Shoemaker CA, Arias CR, Klesius PH (2005). Transmission and detection of *Flavobacterium columnare *in channel catfish *Ictalurus punctatus*. Dis Aquat Organ.

[B9] Arias CR, Welker TL, Shoemaker CA, Abernathy JW, Klesius PH (2004). Genetic fingerprinting of *Flavobacterium columnare *isolates from cultured fish. J Appl Microbiol.

[B10] Darwish AM, Ismaiel AA (2005). Genetic diversity of *Flavobacterium columnare *examined by restriction fragment length polymorphism and sequencing of the 16S ribosomal RNA gene and the 16S-23S rDNA spacer. Mol Cell Probes.

[B11] Schneck JL, Caslake LF (2006). Genetic diversity of *Flavobacterium columnare *isolated from fish collected from warm and cold water. J Fish Dis.

[B12] Suomalainen L-R, Tiirola M, Valtonen ET (2006). Chondroitin AC lyase activity is related to virulence of fish pathogenic *Flavobacterium columnare*. J Fish Dis.

[B13] Thomas-Jinu S, Goodwin AE (2004). Morphological and genetic characteristics of *Flavobacterium columnare *isolates: correlations with virulence in fish. J Fish Dis.

[B14] Altinok I, Grizzle JM (2001). Effects of low salinities on *Flavobacterium columnare *infection of euryhaline and freshwater stenohaline fish. J Fish Dis.

[B15] Bader JA, Shoemaker CA, Klesius PH (2005). Production, characterization and evaluation of virulence of an adhesion defective mutant of *Flavobacterium columnare *produced by beta-lactam selection. Lett Appl Microbiol.

[B16] Decostere A, Haesebrouck F, Turnbull JF, Charlier G (1999). Influence of water quality and temperature on adhesion of high and low virulence *Flavobacterium columnare *strains to isolated gill arches. J Fish Dis.

[B17] Newton J, Wood T, Hartley M (1997). Isolation and partial characterization of extracellular proteases produced by isolates of *Flavobacterium columnare *derived from channel catfish. J Aquat Anim Health.

[B18] Stringer-Roth KM, Yunghans W, Caslake LF (2002). Differences in chondroitin AC lyase activity of *Flavobacterium columnare *isolates. J Fish Dis.

[B19] Tkalec AL, Fink D, Blain F, Zhang-Sun G, Laliberte M, Bennett DC, Gu K, Zimmermann JJF, Su H (2000). Isolation and Expression in *Escherichia coli *of *cslA *and *cslB*, Genes Coding for the Chondroitin Sulfate-Degrading Enzymes Chondroitinase AC and Chondroitinase B, Respectively, from *Flavobacterium heparinum*. Appl Environ Microbiol.

[B20] Bayley D, Rocha E, Smith C (2000). Analysis of *cepA *and other *Bacteroides fragilis *genes reveals a unique promoter structure. FEMS Microbiol Lett.

[B21] Mcbride MJ, Kemp PF (1996). Development of techniques for the genetic manipulation of the gliding bacterium *Cytophaga johnsonae*. J Bacteriol.

[B22] Shoemaker NB, Getty C, Gardner JF, Salyers AA (1986). Tn*4351 *transposes in *Bacteroides *spp. and mediates the integration of plasmid R751 into the *Bacteroides *chromosome. J Bacteriol.

[B23] Li L, Shoemaker N, Salyers A (1995). Location and characteristics of the transfer region of a *Bacteroides *conjugative transposon and regulation of transfer genes. J Bacteriol.

[B24] Alvarez B, Secades P, McBride MJ, Guijarro JA (2004). Development of genetic techniques for the psychrotrophic fish pathogen *Flavobacterium psychrophilum*. Appl Environ Microbiol.

[B25] Chen S, Bagdasarian M, Kaufman MG, Walker ED (2007). Characterization of strong promoters from an environmental *Flavobacterium hibernum *strain by using a green fluorescent protein-based reporter system. Appl Environ Microbiol.

[B26] Soto E, Mauel MJ, Karsi A, Lawrence ML (2008). Genetic and virulence characterization of *Flavobacterium columnare *from channel catfish (*Ictalurus punctatus*). J Appl Microbiol.

[B27] Moyer TR, Hunnicutt DW (2007). Susceptibility of zebra fish *Danio rerio *to infection by *Flavobacterium columnare *and *F. johnsoniae*. Dis Aquat Organ.

[B28] Chen S, Bagdasarian M, Kaufman M, Bates A, Walker E (2007). Mutational analysis of the *ompA *promoter from *Flavobacterium johnsoniae*. J Bacteriol.

[B29] McBride M, Baker S (1996). Development of techniques to genetically manipulate members of the genera *Cytophaga*, *Flavobacterium*, *Flexibacter*, and *Sporocytophaga*. Appl Environ Microbiol.

[B30] Farmer B (2004). Improved methods for the isolation and characterization of *Flavobacterium columnare*.

[B31] Song Y, Fryer J, Rohovec J (1988). Comparison of six media for the cultivation of *Flexibacter columnaris*. Fish Pathology.

[B32] Staroscik A, Nelson D (2008). The influence of salmon surface mucus on the growth of Flavobacterium columnare. J Fish Dis.

[B33] Smith C, Rogers M, McKee M (1992). Heterologous gene expression in *Bacteroides fragilis*. Plasmid.

[B34] Miller W, Lindow S (1997). An improved GFP cloning cassette designed for prokaryotic transcriptional fusions. Gene.

[B35] Xie HX, Nie P, Sun BJ (2004). Characterization of two membrane-associated protease genes obtained from screening out-membrane protein genes of *Flavobacterium columnare *G4. J Fish Dis.

[B36] Agarwal S, Hunnicutt DW, McBride MJ (1997). Cloning and characterization of the *Flavobacterium johnsoniae *(*Cytophaga johnsonae*) gliding motility gene, *gldA*. PNAS.

[B37] Kempf MJ, McBride MJ (2000). Transposon insertions in the *Flavobacterium johnsoniae ftsX *gene disrupt gliding motility and cell division. J Bacteriol.

[B38] Hunnicutt DW, McBride MJ (2001). Cloning and Characterization of the *Flavobacterium johnsoniae *Gliding Motility Genes *gldD *and *gldE*. J Bacteriol.

[B39] Chaudhury A, Smith G (1984). A new class of *Escherichia coli recBC *mutants: implications for the role of RecBC enzyme in homologous recombination. PNAS.

[B40] Clark A (1973). Recombination deficient mutants of *E. coli *and other bacteria. Annu Rev Genet.

[B41] Ivancic-Bace I, Peharec P, Moslavac S, Skrobot N, Salaj-Smic E, Brcic-Kostic K (2003). RecFOR function is required for DNA repair and recombination in a RecA loading-deficient *recB *mutant of *Escherichia coli*. Genetics.

[B42] Duchaud E, Boussaha M, Loux V, Bernardet J, Michel C, Kerouault B, Mondot S, Nicolas P, Bossy R, Caron C, Bessières P, Gibrat JF, Claverol S, Dumetz, Le Hénaff M, Benmansour A (2007). Complete genome sequence of the fish pathogen *Flavobacterium psychrophilum*. Nat Biotechnol.

[B43] Rocha E, Cornet E, Michel B (2005). Comparative and evolutionary analysis of the bacterial homologous recombination systems. PLoS Genet.

[B44] Bertani G (1951). Studies on lysogenesis. I. The mode of phage liberation by lysogenic *Escherichia coli*. J Bacteriol.

[B45] Sambrook J, Fitsch EF, Maniatis T (1989). Molecular cloning: a laboratory manual.

[B46] Eggers C, Caimano M, Radolf J (2004). Analysis of promoter elements involved in the transcriptional initiation of RpoS-dependent *Borrelia burgdorferi *genes. J Bacteriol.

[B47] Braun T, McBride M (2005). *Flavobacterium johnsoniae GldJ *is a lipoprotein that is required for gliding motility. J Bacteriol.

[B48] GSC. http://www.uri.edu/research/gsc/.

[B49] ImageJ. http://rsb.info.nih.gov/ij.

[B50] The GIMP. http://www.gimp.org.

[B51] Marden P, Tunlid A, Malcrona-Friberg K, Odham G, Kjelleberg S Physiological and morphological changes during short term starvation of marine bacteriological isolates. Arch Microbiol.

[B52] Zar J (1999). Biostatistical Analysis.

[B53] Cooper A, Kalinowski A, Shoemaker N, Salyers A (1997). Construction and characterization of a *Bacteroides thetaiotaomicron recA *mutant: transfer of *Bacteroides *integrated conjugative elements is RecA independent. J Bacteriol.

[B54] Ordal EJ, Rucker RR (1944). Pathogenic myxobacteria. Soc Exp Biol Med.

